# Improved detection of RNA foci in *C9orf72* amyotrophic lateral sclerosis post-mortem tissue using BaseScope™ shows a lack of association with cognitive dysfunction

**DOI:** 10.1093/braincomms/fcaa009

**Published:** 2020-01-31

**Authors:** Arpan R Mehta, Bhuvaneish T Selvaraj, Samantha K Barton, Karina McDade, Sharon Abrahams, Siddharthan Chandran, Colin Smith, Jenna M Gregory

**Affiliations:** f1 UK Dementia Research Institute, University of Edinburgh, Edinburgh, UK; f2 Centre for Clinical Brain Sciences, University of Edinburgh, Edinburgh, UK; f3 The Anne Rowling Regenerative Neurology Clinic, University of Edinburgh, Edinburgh, UK; f4 The Euan MacDonald Centre, University of Edinburgh, Edinburgh, UK; f5 Nuffield Department of Clinical Neurosciences, University of Oxford, Oxford, UK; f6 The Florey Institute of Neuroscience and Mental Health, University of Melbourne, Parkville VIC 3010, Australia; f7 School of Philosophy, Psychology and Language Sciences, University of Edinburgh, Edinburgh, UK; f8 Centre for Brain Development and Repair, inStem, Bangalore, India; f9 MRC Centre for Regenerative Medicine, University of Edinburgh, Edinburgh, UK; f10 MRC Edinburgh Brain Bank, Academic Department of Neuropathology, University of Edinburgh, Edinburgh, UK; f11 Edinburgh Pathology, University of Edinburgh, Edinburgh, UK

**Keywords:** sense RNA foci, TDP-43, *C9orf72*, ALS, cognition

## Abstract

The *C9orf72* hexanucleotide repeat expansion is the commonest known genetic mutation in amyotrophic lateral sclerosis. A neuropathological hallmark is the intracellular accumulation of RNA foci. The role that RNA foci play in the pathogenesis of amyotrophic lateral sclerosis is widely debated. Historically, *C9orf72* RNA foci have been identified using *in situ* hybridization. Here, we have implemented BaseScope™, a high-resolution modified *in situ* hybridization technique. We demonstrate that previous studies have underestimated the abundance of RNA foci in neurons and glia. This improved detection allowed us to investigate the abundance, regional distribution and cell type specificity of sense *C9orf72* RNA foci in post-mortem brain and spinal cord tissue of six deeply clinically phenotyped *C9orf72* patients and six age- and sex-matched controls. We find a correlation between RNA foci and the accumulation of transactive response DNA-binding protein of 43 kDa in spinal motor neurons (*r_s_* = 0.93; *P *=* *0.008), but not in glia or cortical motor neurons. We also demonstrate that there is no correlation between the presence of RNA foci and the accumulation of transactive response DNA binding protein of 43 kDa in extra-motor brain regions. Furthermore, there is no association between the presence of RNA foci and cognitive indices. These results highlight the utility of BaseScope™ in the clinicopathological assessment of the role of sense RNA foci in *C9orf72*.

## Teaser

Mehta *et al.* find that the detection of RNA foci in *C9orf72* amyotrophic lateral sclerosis post-mortem tissue using BaseScope™ reveals a lack of association with cognitive dysfunction and improved cell type resolution at low magnification.

## Graphical Abstract

**Graphical Abstract:**
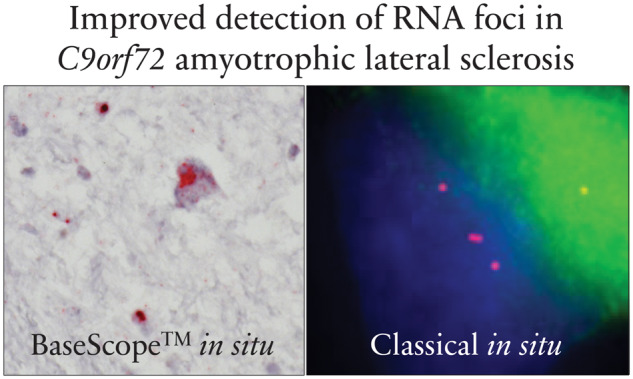

## Introduction

A key post-mortem diagnostic hallmark of most amyotrophic lateral sclerosis (ALS) cases is the presence of intra-cytoplasmic transactive response DNA-binding protein of 43 kDa (TDP-43) aggregates in motor cortex and spinal cord motor neurons and glia, associated with anterior horn cell loss and gliosis (Arai *et al.*, 2006; Neumann *et al.*, 2006). Additionally, we have previously demonstrated widespread cortical TDP-43 (neuronal and glial) aggregation in extra-motor cortical regions linked to executive, language and fluency dysfunction (Gregory *et al.*, 2019).

Familial ALS (fALS) cases account for approximately 10% of all ALS cases; approximately 40% of fALS cases and a proportion of sporadic cases are caused by the *C9orf72* hexanucleotide G_4_C_2_ repeat expansion, making it the commonest known ALS genetic mutation (DeJesus-Hernandez *et al.*, 2011). Such patients typically present with motor symptoms; however, cognitive dysfunction (affecting all domains: fluency, language, executive and behavioural functions) is common, as is a neuropsychiatric presentation (Abrahams *et al.*, 2000; Goldstein and Abrahams, 2013; Strong *et al.*, 2017; Gregory *et al.*, 2019). Intracellular accumulation of sense and antisense RNA foci is a key molecular hallmark in the CNS of *C9orf72* cases. Their precise pathogenic role is widely debated (Vatsavayai *et al.*, 2019); indeed, antisense RNA foci have been shown to exert a greater deleterious effect than sense RNA foci (Gendron *et al.*, 2013; Zu *et al.*, 2013; Cooper-Knock *et al.*, 2015; Aladesuyi Arogundade *et al.*, 2019) and antisense RNA foci, but not sense RNA foci, correlate with TDP-43 aggregation in *C9orf72* motor neurons (Cooper-Knock *et al.*, 2015; Aladesuyi Arogundade *et al.*, 2019).

It therefore remains an open question as to the role of sense RNA foci in extra-motor regions, and whether, if present in these areas, they exert any effect on cognition. Moreover, parsing out the role of RNA foci in non-neuronal cells, and in regions of the brain and spinal cord that are not affected by TDP-43 pathology, remains to be performed. To date, classical *in situ* hybridization (ISH) techniques to investigate the abundance and clinicopathological relevance of RNA foci in human *C9orf72* brain and spinal cord tissue (Gendron *et al.*, 2013; Mizielinska *et al.*, 2013; Cooper-Knock *et al.*, 2015; DeJesus-Hernandez *et al.*, 2017; Aladesuyi Arogundade *et al.*, 2019) have been limited by technical challenges that have prevented these questions from being addressed. First, owing to the substantial autolysis of mRNA in post-mortem samples, ISH probes are impeded in their binding to the target. Secondly, Sudan black tends to be used to quench background autofluorescence, which has the detrimental effect of also quenching signal. Thirdly, there is a lack of resolution and/or sensitivity with consequential inadequate cell type-specific resolution at low magnification, mandating high-resolution imaging of single cells or, in the case of *C9orf72* RNA foci, single nuclei at high resolution; this prevents informative comparative imaging and quantification of large areas of tissue.

BaseScope™ is a modified ISH technique that addresses some of these limitations, allowing for greater sensitivity and specificity, and achieves amplification of mRNA with improved single-cell, single-transcript resolution. The BaseScope™ probes are designed to bind to the mRNA transcript in several sets of probe pairs, allowing for increased sensitivity and specificity. Probe hybridization is followed by the addition of iterative amplification probes, facilitating large numbers of molecules of chromogen (rather than a single molecule in classical ISH) to bind to the probe. This serves to amplify the (partially degraded, autolysed) mRNA transcript signal, thereby permitting the detection of single transcripts at a single-cell level, even at low magnification. Accordingly, this leads to improved spatial resolution, by virtue of being able to image many cells at once, rather than single cells. In addition, the use of chromogen detection with a haematoxylin counterstain to identify cells obviates the need to use Sudan black to quench background autofluorescence and makes it possible to resolve cell type-specific and subcellular localization of RNA foci.

We therefore aimed to use BaseScope™ to visualize *C9orf72* RNA foci and hypothesized that the increased resolution, sensitivity and specificity that this technique confers would allow us to examine whether sense RNA foci are (i) associated with TDP-43 accumulation in extra-motor brain regions and (ii) independently associated with cognitive deficits in these extra-motor brain regions.

## Materials and methods

### Case identification and cognitive profiling

We identified six ALS post-mortem cases with a *C9orf72* hexanucleotide repeat expansion and six age- and sex-matched control cases (with no neurological disorder during life and no significant neuropathology present at post-mortem; Table 1). Each donor patient had undergone neuropsychological testing with the Edinburgh Cognitive and Behavioural ALS Screen (ECAS; https://ecas.psy.ed.ac.uk/; Abrahams *et al.*, 2014). Additionally, all cases had corresponding whole genome sequencing and diagnostic repeat prime polymerase chain reaction, demonstrating pathogenic repeat lengths in the *C9orf72* locus (Leighton *et al.*, 2019). All patients had consented to the use of their data for research (Leighton *et al.*, 2019). The use of human tissue for post-mortem studies has been reviewed and approved by the Edinburgh Brain Bank ethics committee and the Academic and Clinical Central Office for Research and Development medical research ethics committee, in line with the Human Tissue (Scotland) Act 2006.

**Table 1:** Clinical characteristics of cases included in this study

	*C9orf72*-ALS cases	Control cases
Age at death (years)	Median: 60; range: 43–63	Median: 59; range: 41–63
Sex	3 males; 3 females	3 males; 3 females
Disease duration (months)	Median: 44; range: 25–87	N/A
Age at onset (years)	Median: 55; range: 38–60	N/A

### Brain region selection

To build on the recently reported association between antisense RNA foci and TDP-43 accumulation in motor neurons (Cooper-Knock *et al.*, 2015; Aladesuyi Arogundade *et al.*, 2019), we examined motor regions including the anterior horn of the spinal cord and cortical motor neurons in Brodmann area (BA) 4. These areas have previously been examined for the presence of TDP-43 accumulation, and the misfolded protein burden (based on cells demonstrating aberrant phospho-TDP-43 immunoreactivity) was scored from mild to severe (Gregory *et al.*, 2019). Brain regions typically affected in ALS patients experiencing cognitive deficits are the regions associated with language, fluency and executive functions. Thus, we selected a representative brain region associated with language and fluency function (inferior frontal gyrus; BA44/45) and a representative brain region associated with executive function (dorsolateral prefrontal cortex; BA46). We also examined the cerebellum for evidence of RNA foci, since it does not typically have TDP-43 aggregates but, unique to *C9orf72* cases, demonstrates p62-positive inclusions (Al-Sarraj *et al.*, 2011). These aggregates are not associated with cell death or cell loss and co-stain for dipeptide repeat proteins, which result as a direct consequence of the repeat-associated non-AUG (RAN) translation of the *C9orf72* repeat expansion (Gendron *et al.*, 2013).

### BaseScope™ *in situ* hybridization

Brain tissue was processed for BaseScope™ as previously reported (Gregory *et al.*, 2019). We optimized the BaseScope™ technique on control tissue to address probe specificity; C_4_G_2_-repeat probes will also bind to G_4_C_2_ repeats within the DNA, which exist in both control cases and *C9orf72* cases. Deoxyribonuclease (DNase) treatment (optimized specifically to retain haematoxylin counterstaining, but eliminate probe binding to DNA repeats) was carried out at 40°C at a concentration of 800 U/ml for 30 min immediately following the protease III tissue permeabilization step and prior to probe hybridization. This limited probe hybridization to DNA repeats, thereby preventing amplification of background binding to these repeats. Probe hybridization was then performed by incubating the slides with four drops of custom-designed BaseScope™ probe to identify sense *C9orf72* RNA foci, negative control probe (L-2,3-dihydrodipicolinate reductase, *DapB*) or positive control (peptidyl-prolyl cis-trans isomerase, *PPIB*) probe for 2 h at 40°C. The FastRed chromogen incubation step was reduced from 10 min to 2 min. Sections were imaged at ×40 magnification on a NanoZoomer slide scanner (Hamamatsu), and the relative abundance of transcripts was quantified manually by neuropathologists assessing the proportion of cells containing RNA foci and the mean (+/− SD) number of dots/transcripts per cell across 30 cells (10 cells in three randomly generated fields of view).

### Statistical analysis

To assess for associations between sense RNA foci and TDP-43 pathology, we generated a compound product score to reflect the number of foci per cell and the proportion of cells affected by multiplying the mean values from the quantification of these two scores. We then calculated the Spearman’s rank correlation coefficient, inputting the TDP-43 score and the RNA foci product score. Values were considered statistically significant when *P *≤* *0.05. Error bars represent variation within a single case plotted as mean and standard error. Each bar represents a single case.

### Data availability

Data are available from the corresponding authors upon reasonable request.

## Results

### BaseScope™ permits improved detection of sense RNA foci in *C9orf72*-ALS

The mean percentage of cells containing sense RNA foci in motor brain and spinal cord regions was 78.7% for neurons and 24.9% for glia. The mean percentage of cells containing sense RNA foci in extra-motor brain regions was 89.4% for neurons and 46.1% for glia. The mean number of foci per cell in motor brain and spinal cord regions was 2.6 for neurons and 1.1 for glia, and the mean number of foci per cell in extra-motor brain regions was 2.9 for neurons and 1.2 for glia. These data illustrate the substantially improved signal amplification and therefore the sensitivity of this technique when compared with standard ISH techniques (Aladesuyi Arogundade *et al.*, 2019). This improved detection is especially the case at low magnification, as illustrated in the low-magnification images shown in Supplementary Fig. 1, demonstrating clear staining of RNA foci (red dots) without the need to examine a single nucleus at higher magnification. The mean number of foci per cell is comparable to previous estimates in motor neurons, highlighting the robustness of this finding in the light of improved sensitivity (Mizielinska *et al.*, 2013; Aladesuyi Arogundade *et al.*, 2019).

### Sense RNA foci are associated with TDP-43 aggregation in spinal motor neurons but not in motor cortex or spinal cord glia

**Figure 1:**
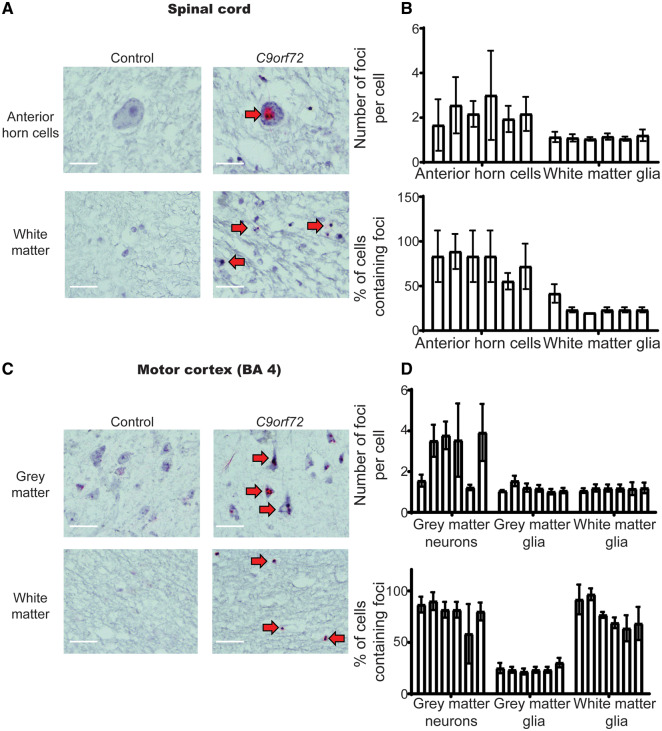
**Motor neuronal—but not glial—RNA is associated with TDP-43 pathology.** **(A)** Representative images of control (left panel) and *C9orf72* (right panel) anterior horn motor neurons (top panel) and glial cells (bottom panel). Images demonstrate negative staining for sense RNA foci in the control tissue and discrete sense RNA foci (red dots) in motor neurons and glia of *C9orf72* cases. Red arrows indicate cells with positive staining, *n* = 6 cases and six age- and sex-matched sudden death controls with no evidence of neurological disease. Scale bars = 40 µm. Residual background binding to DNA (small red dots) can be seen in controls and ALS cases; however, RNA foci are only detected in *C9orf72* cases (large red RNA foci). **(B)** Graph quantifying number of RNA foci per cell and the proportion of cells that contain RNA foci in the anterior horn of the spinal cord; *n* = 6 cases and six age- and sex-matched sudden death controls with no evidence of neurological disease. Each bar represents a single case, error bars demonstrate variation between regions assessed within the pathological material analysed for each case. **(C)** Representative images of control (left panel) and *C9orf72* (right panel) cortical motor neurons (top panel) and cortical glial cells (bottom panel). Images demonstrate negative staining for sense RNA foci in the control tissue and discrete sense RNA foci (red dots) in motor neurons and glia of *C9orf72* cases. Red arrows indicate cells with positive staining, n = 6 cases and six age- and sex-matched sudden death controls with no evidence of neurological disease. Scale bars = 40 µm. Residual background binding to DNA (small red dots) can be seen in controls and ALS cases; however, RNA foci are only detected in *C9orf72* cases (large red RNA foci). **(D)** Graph quantifying number of RNA foci per cell and the proportion of cells that contain RNA foci in the motor cortex; *n* = 6 cases and six age- and sex-matched sudden death controls with no evidence of neurological disease. Each bar represents a single case, error bars demonstrate variation between regions assessed within the pathological material analysed for each case.

**Figure 2:**
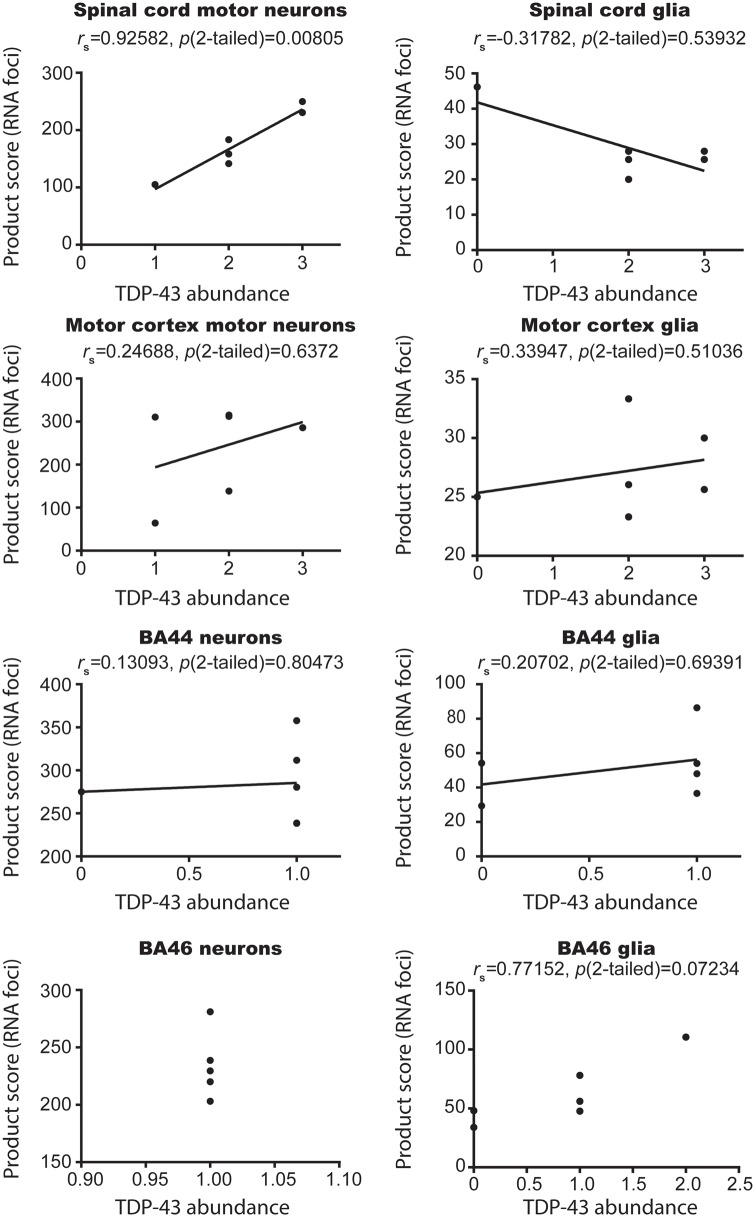
**Statistically significant correlation between TDP-43 abundance and RNA foci in spinal cord of *C9orf72* patients.** Scatter plots of TDP-43 abundance on the abscissa and RNA foci product score (a function of number of foci and percentage of cells affected) on the ordinate. Each point on the graph is an individual case. *r_s_* values were calculated using Spearman’s correlation, and values were considered significant when *P* < 0.05. Data demonstrate that a correlation between RNA foci and TDP-43 exists only in motor neurons of the spinal cord in *C9orf72* patients.

Previously, it has been shown that antisense RNA foci are associated with TDP-43 pathology in spinal motor neurons of *C9orf72*-ALS cases (Cooper-Knock J *et al.*, 2015; Aladesuyi Arogundade *et al.*, 2019). Here, all six cases demonstrated TDP-43 pathology in the motor neurons of the spinal cord and motor cortex, and also demonstrated concomitant neuronal sense RNA foci (Table 2; Fig. 1). In contrast, the association between TDP-43 aggregation and the presence of sense RNA foci was not apparent in glial cells in the same regions. Case 1 had no evidence of glial TDP-43 aggregation, but a similar burden of sense RNA foci to the other cases that did demonstrate TDP-43 pathology. Moreover, the examination of the remaining five cases revealed trends in expression that also support this finding; Cases 2 and 4 appeared to have the most abundant neuronal TDP-43 pathology and correspondingly the highest abundance of neuronal sense RNA foci in both the spinal cord and the motor cortex. Similarly, Case 5 had the least abundant neuronal TDP-43 pathology and correspondingly the least abundant levels of neuronal sense RNA foci. This association, with respect to trends in expression of TDP-43 and the presence of sense RNA foci, did not hold true for the glial cells in the motor cortex and spinal cord. To assess the association between the abundance of RNA foci and TDP-43 aggregation in further detail, we next calculated the Spearman’s rank correlation coefficients to compare TDP-43 abundance to a product score comprised of the percentage of RNA foci positive cells and the number of foci per cell for each of the brain regions and spinal cord segments examined for both glial and neuronal cell populations. This test demonstrated a significant correlation for spinal motor neurons (*r_s_* = 0.93; *P *=* *0.008), but not for spinal cord glia (*r_s_* = −0.32; *P *=* *0.54), motor cortex neurons (*r_s_* = 0.25; *P *=* *0.64) or motor cortex glia (*r_s_* = 0.34; *P *=* *0.51; Fig. 2).

**Table 2:** Scoring of regional TDP-43 pathology

Case	Cell type	BA4	Spinal cord	BA44/45	BA46	Cerebellum
1	Neuron	2	2	1	1	0
	Glia	0	0	0	0	0
2	Neuron	2	3	1	1	0
	Glia	3	3	1	1	0
3	Neuron	1	2	0	1	0
	Glia	2	2	0	0	0
4	Neuron	3	3	1	1	0
	Glia	3	3	1	1	0
5	Neuron	1	1	1	1	0
	Glia	2	2	1	2	0
6	Neuron	2	2	1	1	0
	Glia	2	2	1	1	0

The table summarizes pathological TDP-43 staining using a phospho-TDP-43 antibody. Scoring of TDP-43 pathology is based on the semi-quantitative scoring published previously (Gregory *et al*., 2019): 0—no evidence of TDP-43 pathology, 1—mild pathology, 2—moderate pathology and 3—severe pathology. Neuronal and glial pathology have been quantified individually.

### Sense RNA foci are not associated with TDP-43 aggregation or clinical manifestations in extra-motor brain regions

**Figure 3:**
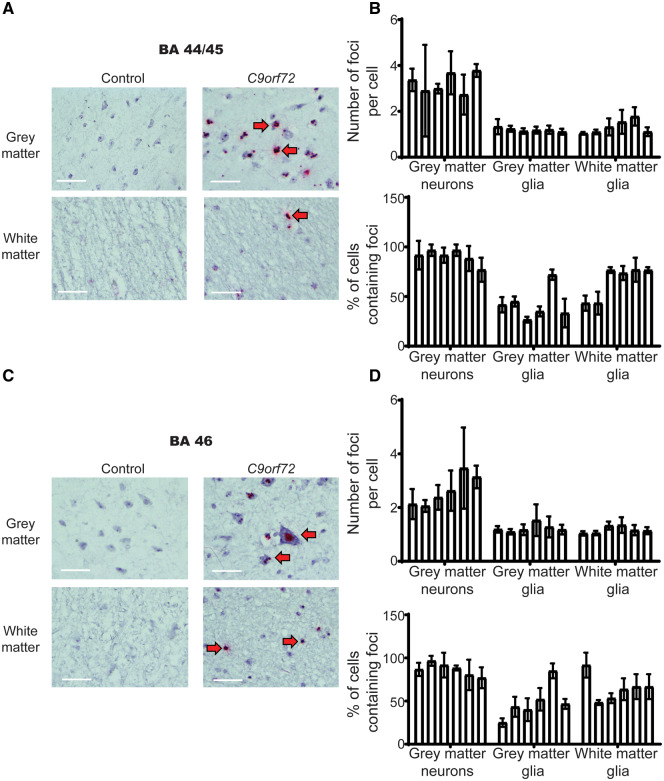
**Absence of association between presence or abundance of RNA foci and clinical manifestations or TDP-43 pathology in extra-motor regions.** **(A)** Representative images of control (left panel) and *C9orf72* (right panel) neurons of BA44 brain region associated with language and fluency dysfunction (top panel) and glial cells (bottom panel). Images demonstrate negative staining for sense RNA foci in the control tissue and discrete sense RNA foci (red dots) in neurons and glia of *C9orf72* cases. Red arrows indicate cells with positive staining, *n* = 6 cases and six age- and sex-matched sudden death controls with no evidence of neurological disease. Scale bars = 40 µm. Residual background binding to DNA (small red dots) can be seen in controls and ALS cases; however, RNA foci are only detected in *C9orf72* cases (large red RNA foci). **(B)** Graph quantifying number of RNA foci per cell and the proportion of cells that contain RNA foci in BA44; *n* = 6 cases and six age- and sex-matched sudden death controls with no evidence of neurological disease. Each bar represents a single case, error bars demonstrate variation between regions assessed within the pathological material analysed for each case. **(C)** Representative images of control (left panel) and *C9orf72* (right panel) neurons of BA46 brain region associated with executive dysfunction (top panel) and cortical glial cells (bottom panel). Images demonstrate negative staining for sense RNA foci in the control tissue and discrete sense RNA foci (red dots) in neurons and glia of *C9orf72* cases. Red arrows indicate cells with positive staining, *n* = 6 cases and six age- and sex-matched sudden death controls with no evidence of neurological disease. Scale bars = 40 µm. Residual background binding to DNA (small red dots) can be seen in controls and ALS cases, however RNA foci are only detected in *C9orf72* cases (large red RNA foci). **(D)** Graph quantifying number of RNA foci per cell and the proportion of cells that contain RNA foci in BA46; *n* = 6 cases and six age- and sex-matched sudden death controls with no evidence of neurological disease. Each bar represents a single case, error bars demonstrate variation between regions assessed within the pathological material analysed for each case.

**Figure 4:**
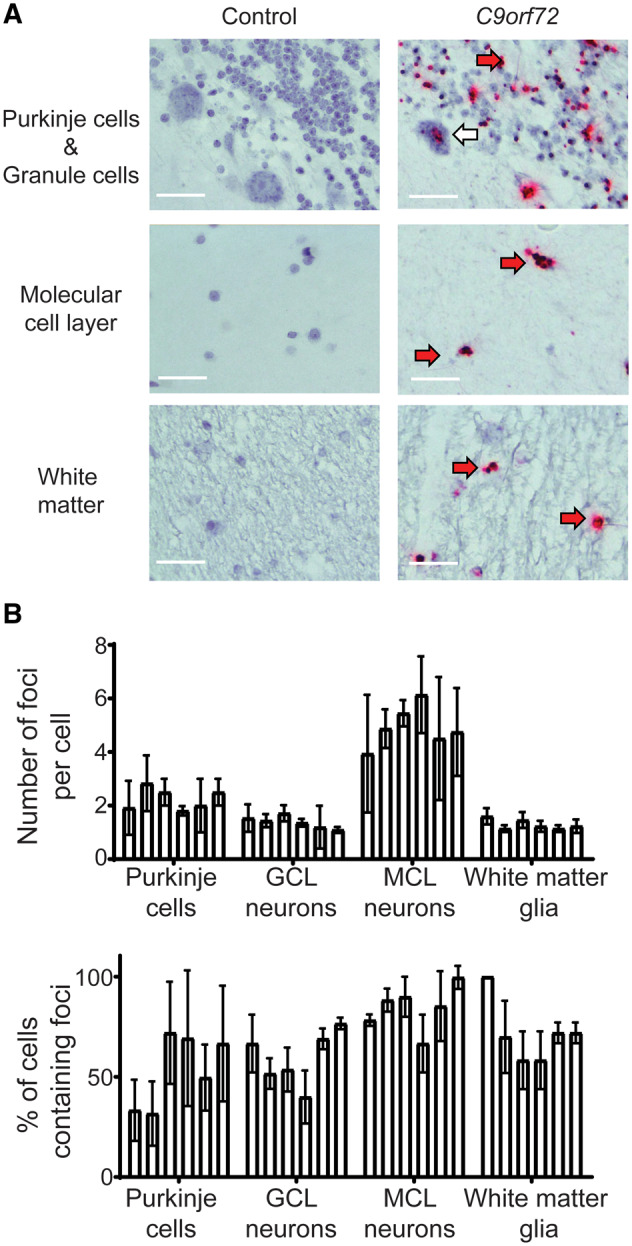
**Absence of association between presence or abundance of RNA foci and clinical manifestations or TDP-43 pathology extra-motor regions.** **(A)** Representative images of control (left panel) and *C9orf72* (right panel) cerebellar Purkinje and granule cells (top panel; GCL), molecular cell layer neurons (middle panel; MCL) and white matter glial cells (bottom panel). Images demonstrate negative staining for sense RNA foci in the control tissue and discrete sense RNA foci (red dots) in cells of *C9orf72* cases. Red arrows indicate cells with positive staining, *n* = 6 cases and six age- and sex-matched sudden death controls with no evidence of neurological disease. Scale bars = 40 µm. **(B)** Graph quantifying number of RNA foci per cell and the proportion of cells that contain RNA foci in the cerebellum; n = 6 cases and six age- and sex-matched sudden death controls with no evidence of neurological disease. Each bar represents a single case, error bars demonstrate variation between regions assessed within the pathological material analysed for each case.

Given the association between TDP-43 pathology and RNA foci in spinal cord motor neurons, and the association between TDP-43 and cognitive deficits in ALS (Henstridge *et al.*, 2017; Gregory *et al.*, 2019), we next sought to examine the relationship between TDP-43 and sense RNA foci in extra-motor brain regions responsible for cognition. Importantly, all six cases examined in this study had cognitive testing during life using the same screening tool—the ECAS. We have previously documented the TDP-43 distribution of the six cases assessed in this study (Gregory *et al.*, 2019), demonstrating that the presence of TDP-43 pathology in brain regions associated with executive function, language and fluency is associated with cognitive deficits. None of the cases examined in this study met the criteria for a diagnosis of frontotemporal dementia or had behavioural symptoms. Three cases had mild language deficits, one case had mild executive dysfunction and two cases had normal cognitive function (Table 3). Despite having marked differences in extra-motor clinical manifestations (differentially affected subdomains of cognition), all cases had a similar regional distribution and abundance of RNA foci in extra-motor brain regions (Fig. 3). Spearman’s rank correlation coefficient demonstrated no significant correlation between sense RNA foci in BA44 neurons (*r_s_* = 0.13093, *P *=* *0.80473) or glia (*r_s_* = 0.20702, *P *=* *0.69391) or BA46 glia (*r_s_* = 0.77152, *P *=* *0.07234; Fig. 2). It was not possible to calculate a correlation coefficient for BA46 neurons, since all of the TDP-43 values were the same. Furthermore, we found evidence of abundant RNA foci in all cell types of the cerebellum, where there is an absence of any TDP-43 pathology (Fig. 4).

**Table 3:** Cognitive profile of the cases included in this study

Case	ECAS total	Executive	Fluency	Language	Disease duration (months)
1	102	**31**	18	27	97
2	112	39	20	**26**	58
3	102	35	18	**23**	58
4	100	36	18	**20**	60
5	114	39	16	28	109
6	118	40	18	28	33

The table summarizes cognitive scores for each of the cases included in this study. Scores reflect total ECAS (which are not significantly impaired for all individuals) and subdomain scores. Boxes highlighted in bold are indicative of a score that falls below the normal threshold according to published cut-offs. Therefore, bold-highlighted boxes indicate a mild cognitive deficit in the specified ECAS subdomain.

## Discussion and Conclusions

Using a bespoke BaseScope™ protocol, we build on previous studies that have implemented classical *in situ* techniques. We show, for the first time, that sense RNA foci are associated with TDP-43 pathology in spinal motor neurons of *C9orf72* cases, in contrast to previous reports (Cooper-Knock J *et al.*, 2015; Aladesuyi Arogundade *et al.*, 2019). The advantage of our novel methodology is that it affords appreciably higher sensitivity and specificity when compared with classical *in situ* hybridization, especially at low magnification (Supplementary Fig. 1), resulting in greater identification of the burden of sense RNA foci than was previously possible, including an examination for regional and cell type specificity (Gendron *et al.*, 2013; Mizielinska *et al.*, 2013; Cooper-Knock *et al.*, 2015; DeJesus-Hernandez *et al.*, 2017; Aladesuyi Arogundade *et al.*, 2019).

Using this technique, we demonstrate that previous estimates of sense RNA foci, with a similar sample size of *C9orf72* cases (*n *=* *5; Aladesuyi Arogundade *et al.*, 2019) are underestimates. Our data demonstrate that the mean percentage of cells containing sense RNA foci is 78.7% (cf. 16%) for neurons and 24.9% (cf. 1%) for glia in motor regions and 89.4% for neurons and 46.1% for glia in extra-motor regions.

Thus, we show the burden of sense RNA foci on glial pathology and that, unlike that for motor neurons, sense RNA foci present in glial cells both in the motor cortex and the spinal cord do not associate with TDP-43 pathology. We also show, for the first time, that there is no association between TDP-43 pathology and sense RNA foci in extra-motor brain regions in neurons, and we demonstrate this same finding also in glial cell populations. Indeed, we identified sense RNA foci of varying abundance in all extra-motor brain regions, irrespective of underlying TDP-43 pathology. Our cases had been deeply clinically phenotyped in life and, as such, we examined the influence of sense RNA foci on cognitive function using the ECAS, designed for, and validated in, ALS (Abrahams *et al.*, 2014; Niven *et al.*, 2015). Accordingly, we show, for the first time, through analysis of cell type-specific sense RNA foci staining, that glial cells and neuronal cells examined in all brain regions contained RNA foci, the abundance of which did not vary with differing clinical phenotypes, for example: cognition, disease duration or extent of regional involvement. Notably, this is also the case for TDP-43 pathology. While the presence of TDP-43 proteinopathy is associated with clinical manifestations of both FTD and ALS, the abundance of TDP-43 pathology is not related to clinical severity (Cykowski *et al.*, 2017). We cannot rule out the possibility of there having been mismatch cases (defined as cases with sense RNA foci but no clinical manifestations as we have seen previously with TDP-43 pathology; Gregory *et al.*, 2019).

Taken together, we demonstrate the utility of a sensitive and specific method for easily identifying sense RNA foci in *C9orf72*-ALS brain and spinal cord tissue, concluding that there is no association between the abundance or presence of sense RNA foci in extra-motor brain regions of patients with *C9orf72*-ALS and their cognitive ability.

## Study limitations

A limitation of studies assessing post-mortem samples that are associated with cognitive data obtained via the same cognitive screening tool is the small sample size. We identified six cases for examination in this study; a larger sample size would afford the power to detect more subtle clinicopathological correlations. Notwithstanding this small sample size, we were able to demonstrate a statistically significant correlation between sense RNA foci and TDP-43 aggregation in spinal motor neurons. Of course, we cannot exclude the possibility that in other brain regions there may be more subtle correlations for which we did not have the power to detect.

Furthermore, we appreciate that it would be useful to demonstrate TDP-43 aggregation and RNA foci in the same cells in a single tissue section; however, likely due to the large size of the RNA foci and amplification cassette used in the BaseScope™ technique, the TDP-43 antibodies are not able to enter the cytoplasm due to steric hindrance, which is why we chose to score the pathologies separately, in line with previous studies. This is a key area for development in molecular pathology, which would improve the accuracy of clinicopathological correlations such as these.

## Supplementary Material

fcaa009_Supplementary_Data

## Abbreviations

ALS =: amyotrophic lateral sclerosis
BA =: Brodmann area
*C9orf72* =: chromosome 9 open reading frame 72
DapB =: L-2,3-dihydrodipicolinate reductase
DNase =: deoxyribonuclease
ECAS =: Edinburgh Cognitive and Behavioural Screen
fALS =: familial amyotrophic lateral sclerosis
ISH =: *in situ* hybridization
mRNA =: messenger RNA
PPIB =: peptidyl-prolyl cis-trans isomerase B
RAN =: repeat-associated non-AUG
TDP-43 =: transactive response DNA-binding protein of 43 kDa
