# Corrigendum

**DOI:** 10.1093/braincomms/fcaa083

**Published:** 2020-07-06

Arpan R. Mehta, Bhuvaneish T. Selvaraj, Samantha K. Barton, Karina McDade, Sharon Abrahams, Siddharthan Chandran, Colin Smith, and Jenna M. Gregory. Improved detection of RNA foci in *C9orf72* amyotrophic lateral sclerosis post-mortem tissue using BaseScope^TM^ shows a lack of association with cognitive dysfunction. Brain Communications 2020. doi:10.1093/braincomms/fcaa009.

The authors were gifted with product: “BaseScope™ Probe - BA-GGGGCCn-3zz-st” from Advanced Cell Diagnostics (Cat Code 704181) in this study. The authors were of the understanding that a probe labelled as “G_4_C_2_-probe” would target C_4_G_2_, namely antisense foci (*e.g.*, as per Lagier-Tourenne *et al.*, 2013 *PNAS*). However, the authors have now been informed by Advanced Cell Diagnostics that, upon their internal review, the probes bind sense *C9orf72* RNA foci. The authors wish to inform readers that the findings in this paper remain valid, save for the word “antisense”, which, when referring to primary data, should read “sense”. The authors have amended their publication accordingly.

