# Supplementary material for: Improved detection of RNA foci in C9orf72 amyotrophic lateral sclerosis post-mortem tissue using BaseScope™ shows a lack of association with cognitive dysfunction
Source: Brain Commun. 2020 Jan 31;2(1):fcaa009. doi: 10.1093/braincomms/fcaa009 (PMC7099934; doi:10.1093/braincomms/fcaa009)
Supplement: fcaa009_Supplementary_Data [file fcaa009_supplementary_data.pdf]

Supplementary Figure 1 Mehta *et al.*

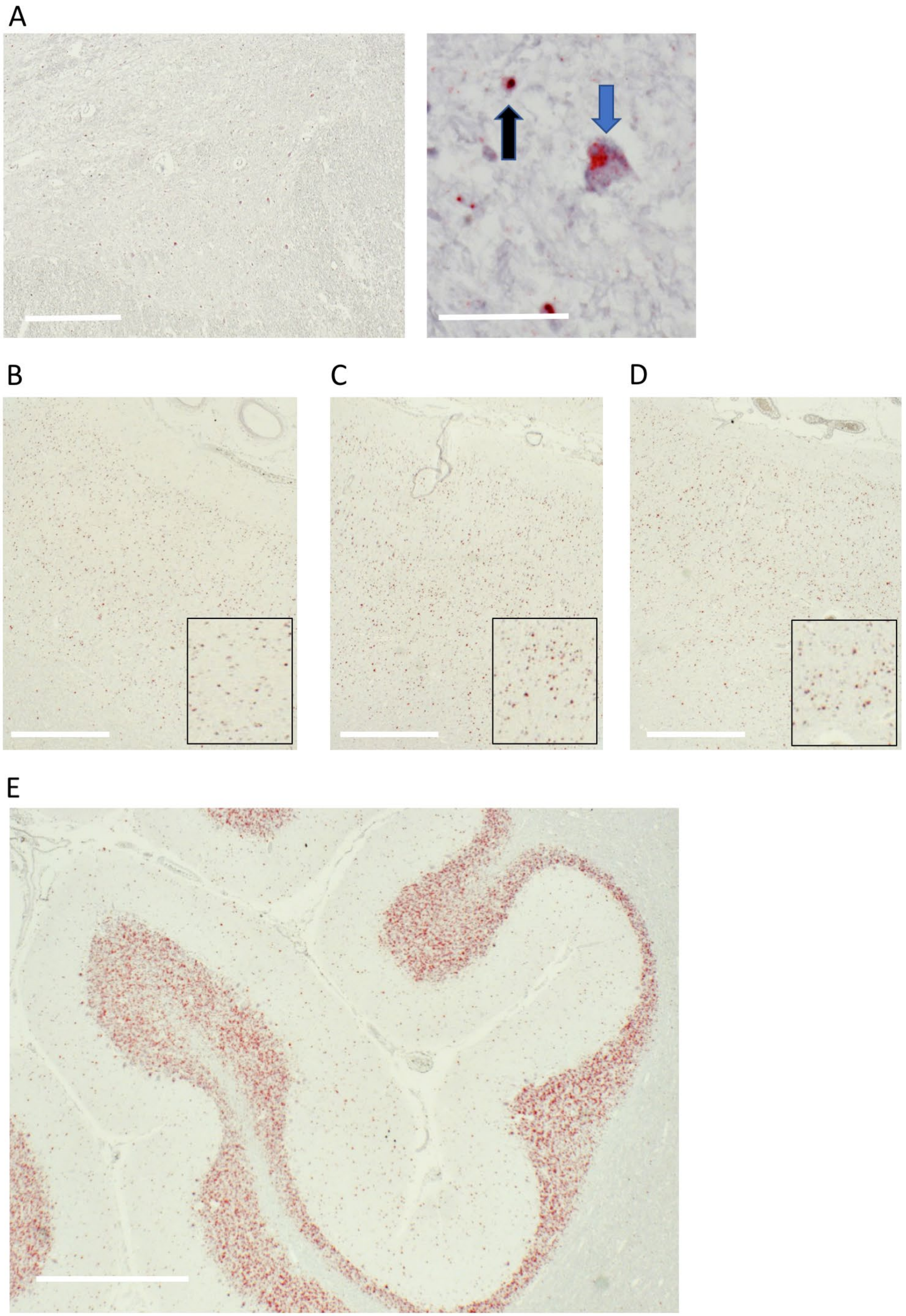

Supplementary Figure 1. BaseScope™ images taken at low magnification (2x and 4x) demonstrate improved detection of RNA foci

Representative images of **A**. Anterior horn of spinal cord (*left*: low magnification [4x], *right*: high magnification [40x]). Scale bar = 0.5 mm on left and 50 µm on right. Blue arrow points to a motor neuron and black arrow points to a glial cell. **B**. Motor cortex; **C**. BA44/45; **D.** BA46 and **E**. Cerebellum (note particularly prominent staining in the granular cell layer) of *C9orf72*-ALS patients, demonstrating clearly visible RNA foci at low magnification. All images of cortex (B-E) taken at 2x magnification. Scale bar = 1 mm. Note reduced staining of haematoxylin counterstain resulting from DNase pretreatment.
